# Production and Evaluation of an Inactivated Adjuvanted Vaccine against Canine Parvovirus in Morocco

**DOI:** 10.3390/vaccines12090995

**Published:** 2024-08-30

**Authors:** Ghizlane Sebbar, Safae El Azhari, Mourad Drifa, Said Mouhri, Mustapha Hammouchi, Hajar Moudhich, Chafiqa Loutfi, Farid Amraoui

**Affiliations:** Société de Productions des Produits Biologiques & Vétérinaires, Avenue Hassan II, Rabat 10051, Morocco

**Keywords:** canine parvovirus, puppies, real-time PCR, isolation, inactivated adjuvanted vaccine, Morocco

## Abstract

The study conducted in Morocco focused on addressing the challenges posed by canine parvovirus (CPV-2) through comprehensive research, vaccine development, and efficacy assessment. Through real-time PCR screening and genotyping, CPV-2 variants were identified circulating in the region. An inactivated vaccine, derived from a CPV-2 strain isolated from a symptomatic dog, was produced and evaluated for safety and efficacy. The vaccine, from the strain named “CaPV M/3-2022”, demonstrated safety in vaccinated puppies, with no adverse reactions observed during the trial period. Efficacy trials showed that vaccinated puppies remained healthy and exhibited lower viral excretion post-challenge compared to unvaccinated controls. These results indicate that the vaccine effectively protects against illness related to CPV-2 and reduces viral shedding. The study provides valuable insights into CPV-2 epidemiology in Morocco, offers a promising vaccine solution, and underscores the importance of vaccination in controlling CPV-2 outbreaks and protecting canine health.

## 1. Introduction

“CPV-2” typically refers to Canine Parvovirus-2, which is a highly contagious viral illness that affects dogs, especially puppies. It can cause severe gastrointestinal symptoms and can be life-threatening if not treated promptly period. It is caused by canine parvovirus, a single-stranded DNA virus categorized within the *Protoparvovirus* genus, specifically the carnivore *Protoparvovirus* 1 species [1]. CPV-2 leads to hemorrhagic gastroenteritis in canines and various other species. The original CPV-2 strain emerged in the USA during a 1978 epidemic of gastroenteritis and subsequently spread globally. CPV-2 was swiftly succeeded by its variants: CPV-2a (1980), CPV-2b (1984), and CPV-2c (2000) [2].

Research conducted in Morocco [3] has confirmed the presence of canine parvovirus using the Snap Parvo-Ag^®^ rapid test (IDEXX, Westbrook, ME, USA), Hemagglutination test, and Hemagglutination inhibition. Sequencing work by Lafquih [4] revealed the co-circulation of the original CPV-2 strain and the CPV-2c variant. Canine parvovirus was successfully isolated in cell culture from MDCK lines by Amrani [5] in 2013. Additionally, Mellor [6] developed an adjuvanted inactivated vaccine based on a Moroccan parvoviral strain, while Achaoui [7] highlighted the coagglutination test for diagnosing canine parvovirus. Despite vaccination efforts, canine parvovirus remains a significant concern in Morocco due to the high number of vaccinated dogs succumbing to the disease. This is largely due to the lack of means for early and rapid diagnosis and uncertainty regarding the efficacy of vaccines used. Thus, there is a pressing need for cost-effective, rapid diagnostic tools to facilitate timely treatment and prevent fatal complications. Furthermore, the parvovirus vaccines currently used in Morocco are based on the original CPV-2 strain, with unproven efficacy.

Several authors have demonstrated limited cross-protection between CPV-2 variants [8,9] and suggested incorporating variant strains into vaccines. Therefore, it is essential to identify circulating variants in Morocco to assess cross-protection and develop more efficient, accessible, and affordable vaccines based on circulating strains.

This study aims to confirm and type all variants circulating in Morocco using specific real-time PCR on 11 samples collected in 2021. Subsequently, isolation on MDCK cells was attempted to select a candidate strain for producing and evaluating an inactivated vaccine derived from the Moroccan canine parvovirus strain.

## 2. Materials and Methods

### 2.1. Samples and Preparation

The eleven specimens utilized in this investigation were obtained from puppies exhibiting symptoms of gastroenteritis and suspected canine parvovirus infection during the month of May 2021. Rectal swabs were immersed in 2 mL of PBS (phosphate-buffered saline) and macerated for one hour before being homogenized.

### 2.2. DNA Extraction

DNA extraction from rectal swabs was conducted using the Venor GeM Sample Preparation kit (Minerva Biolabs, Berlin, Germany) following the manufacturer’s guidelines.

### 2.3. Parvovirus Detection by Real-Time PCR

Real-time polymerase chain reaction (PCR) was performed using the Superscript III Platinum One-Step qRT-PCR kit (Life Technologies, Carlsbad, CA, USA) following the manufacturer’s instructions. A volume of 5 µL of DNA was utilized in a total reaction volume of 20 µL. The primers and probe employed in this study were designed by [10]. The assay was conducted on AriaMx real-time PCR system (Agilent technologies, Santa Clara, CA, USA) with the following cycling profile: polymerase activation at 95 °C for 2 min, followed by 40 cycles of amplification (95 °C for 1 min, 60 °C for 1 min, and 72 °C for 20 s).

### 2.4. Genotyping by Real-Time PCR

Molecular typing was conducted using the Superscript III Platinum One-Step qRT-PCR kit (Life Technologies, Carlsbad, CA, USA) in accordance with the manufacturer’s instructions. Specific primers and probes (Table 1) targeting CPV-2a, CPV-2b, and CPV-2c were employed, as described by [11]. A volume of 5 µL of DNA was utilized in a total reaction volume of 20 µL. The assay was performed on an AriaMx real-time PCR system (Agilent technologies, Santa Clara, CA, USA) with the following cycling profile: polymerase activation at 95 °C for 2 min, followed by 40 cycles of amplification (95 °C for 1 min, 60 °C for 1 min, and 72 °C for 20 s).

### 2.5. Isolation

The strain was initially isolated on a Crandell–Rees Feline Kidney (CRFK) cell and subsequently adapted on Madin–Darby Canine Kidney (MDCK) cells through a total of six passages; these passages constitute the primary viral strain.

In the isolation studies, Madin–Darby Canine Kidney (MDCK) cell lines were utilized. Samples exhibiting a high threshold cycle (Ct < 25) value in polymerase chain reaction (PCR) which indicated a significant amount of virus were filtered using a 0.22 µm membrane filter (Millipore, Bangalore, India), and the filtrates were then employed in the isolation studies. Each sample underwent five passages for virus isolation. The infected cell monolayers were harvested on the fourth day post-inoculation, regardless of cytopathic effects (CPE), through three cycles of alternate freezing and thawing. The virus suspension was clarified by centrifugation at 6000 rpm for 15 min in a refrigerated centrifuge, and the supernatant was subsequently frozen at −40 °C for further use [12].

### 2.6. Inactivated Vaccine Production

The “Canivax-parvo” vaccine’s active ingredient is produced through propagation of the MDCK cell line in single-use Multitrays, followed by inoculation with the seed virus and incubation in roller flasks. After 24 h, the medium is changed, and the cells are left to incubate for 3 days. The viral harvest undergoes quality control sampling before being inactivated with beta-propiolactone overnight at 2–8 °C.

MDCK cells are meticulously examined to ensure their sterility from bacterial, fungal, and mycoplasma contaminants, as well as their virological purity. This comprehensive assessment involves testing for cytopathic and hemadsorbent exogenous viruses at various stages, including the initial stock (MCS), primary stock, and working stock (WCS), in accordance with European Pharmacopoeia monograph No. 50204. Furthermore, throughout the cell amplification phases required for active ingredient production, ongoing checks for bacterial and fungal sterility are conducted at each subculture level to maintain quality standards.

### 2.7. Evaluation Protocol

The inactivated vaccine is subjected to rigorous assessment to determine both its safety and efficacy.

#### 2.7.1. Safety

The safety study for “Canivax-parvo” vaccine was carried out in accordance with European Pharmacopoeia monograph 0795, edition 10.0.

Eight healthy seven-week-old local breed puppies, dewormed and stabilized, were kept at BIOPHARMA pet store for two weeks. They tested negative for CPV-2 before vaccination. Prior to vaccination, a veterinarian confirmed their good health. The primary vaccination involved a 1 mL subcutaneous dose on the first day, followed by another dose three weeks later. Daily clinical examinations and temperature recordings were conducted for 14 days post-vaccination. Blood samples were taken every 4 days to check for leukopenia. Clinical examinations assessed general condition, behavior, injection site reaction, feces consistency, mucous membranes, breathing, cardiac function, dehydration, and any abnormal signs possibly due to vaccination. Monitoring was recorded on the registration form provided for this purpose.

#### 2.7.2. Efficacy

Efficacy trials were conducted following the guidelines and recommendations outlined in the European Pharmacopoeia monograph 0795 edition 10.0.

Two trials were conducted: the vaccination-virulent test and the evaluation of seroconversion in vaccinated puppies and guinea pigs, adhering to the protocol outlined by the European Pharmacopoeia.

***vaccination-virulent test***

Seven puppies (eight weeks old) were divided into two groups: five received “Canivax-parvo” vaccine, while two served as controls. After vaccination, all puppies were monitored daily. After 21 days, both groups were challenged with the test virus and observed for 14 days. Blood samples and rectal swabs were collected for analysis. The vaccine’s effectiveness was determined based on the absence of clinical signs and low viral shedding compared to unvaccinated puppies.

***Evaluation of post-vaccination seroconversion***

- In the target species, seroconversion was evaluated by detecting hemagglutination-inhibiting antibodies in the serum of five vaccinated puppies before the challenge on days 7–14 and day 21. The inhibition of hemagglutination was observed using pig red blood cells at a temperature of 4 °C.
- Guinea pigs were chosen as the animal model to assess the serological response following vaccination. Eight guinea pigs received two subcutaneous injections spaced 14 days apart, each administered at half the recommended dose (0.5 mL). Blood samples were collected on the 14th day after the second injection. The serum was separated by centrifugation and stored at −20 °C until further use. Compliance of the vaccine is determined if the average hemagglutination-inhibiting antibody titer obtained from the eight guinea pigs is at least 1/80.

## 3. Results

### 3.1. Screening and Genotyping Real-Time PCR

Analysis of eleven swabs taken by real-time PCR showed that all eleven samples (100%) were positive. The breakdown of these positive results is as follows: four samples were 2b, five were 2c, and two were 2bc.

The result affirms the presence of genotypes 2b and 2c in circulation with interesting rates (Ct between 11 to 32). Additionally, instances of detecting co-infection with both 2b and 2c genotypes is confirmed. The screening findings are outlined in the subsequent Table 2.

### 3.2. Isolation

The “CaPV M/3-2022” vaccine strain, which is a CPV-2-bc incorporated into “Canivax-parvo”, originates from an indigenous strain obtained from a rectal swab collected from a Malinois exhibiting symptoms of canine parvovirosis.

### 3.3. Evaluation of Inactivated Vaccine Production

#### 3.3.1. Safety

Daily observation of puppies vaccinated with “Canivax-parvo” showed no particular or unusual clinical indications in animals administered with the prescribed dosage via the subcutaneous route (two injections spaced three weeks apart). Over the 35-day observation period, all eight vaccinated puppies remained lively and in good health.

**Figure 1:**
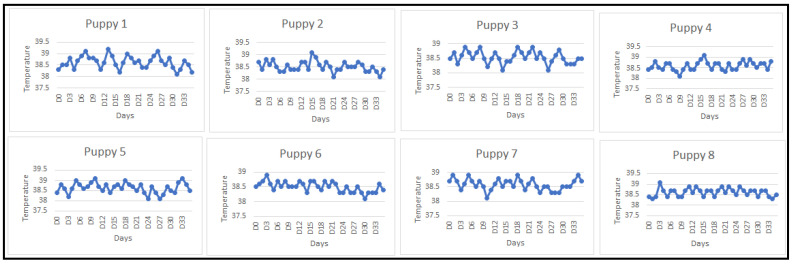
Body temperature monitoring curves. D: day.

The daily rectal temperature measurements of the vaccinated puppies revealed no instances of hyperthermia within the test group. Throughout the observation period, the temperatures remained within the normal range for canine species, typically between 38 °C and 39 °C. Hyperthermia, defined as temperatures of 40.5 °C and above, was not observed. The graph illustrating the daily monitoring of body temperature is presented below (Figure 1).

The assessment of local reactions during the administration of both the initial and booster vaccinations showed no adverse effects.

To evaluate the safety of the “Canivax-parvo” vaccine and confirm its harmlessness, leukopenia, a known syndrome associated with canine parvovirus (CPV-2), was examined using fresh blood samples collected in EDTA tubes. This test provided insights into the inactivation of the virus present in the vaccine formulation and its safety. The results demonstrated that the “Canivax-parvo” vaccine did not induce leukopenia in the vaccinated puppies. The white blood cell count remained within the normal range recognized for canine species, which typically ranges from 6000/mm^3^ to 17,000/mm^3^.

**Figure 2:**
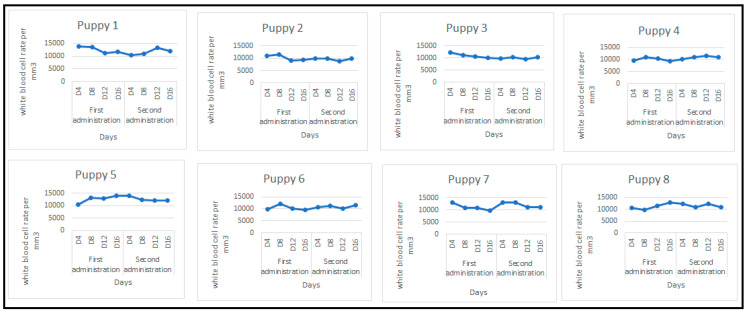
White blood cell rate per mm^3^ for each puppy. D: day.

The following figure (Figure 2) presents the white blood cell count (WBC) per cubic millimeter (mm^3^) for each puppy recorded during the observation period.

#### 3.3.2. Efficacy

Daily monitoring of both vaccinated animals and control groups revealed no clinical abnormalities in the vaccinated puppies and guinea pigs, thus confirming the safety of the vaccine used.

Following the challenge, clinical observations indicated that the five puppies vaccinated with the “Canivax-parvo” vaccine showed no signs of canine parvovirus-related illness. These puppies remained lively and healthy throughout the trial period. Conversely, the two control puppies exhibited specific disease symptoms starting from day 6 post-challenge. These symptoms included lethargy, elevated body temperature (Table 3), and acute gastroenteritis with bloody diarrhea. Despite the administration of symptomatic treatment upon the onset of diarrhea, the condition of these puppies deteriorated by days 9 and 10. Unfortunately, one of the control puppies succumbed to complications on day 9, while the second one passed away on day 11.

Blood counts were conducted immediately after sampling through fresh blood smears. Samples were obtained on days D0, D4, D7, D10, and D14 post-vaccination, and on days D4, D7, D10, and D14 post-challenge. The normal range of white blood cells (leukocytes) in dogs falls between 6000/mm^3^ and 17,000/mm^3^; when this count drops below normal, it is termed leukopenia. Our findings revealed that the vaccine did not induce leukopenia, as the leukocyte count remained within the normal range post-vaccination. There was no discernible difference compared to unvaccinated puppies. Following the challenge, the leukocyte levels remained normal in vaccinated subjects, unlike non-vaccinated ones where leukopenia was observed (Table 4).

Post-challenge viral excretion was also evaluated (Table 5). PCR results indicated that viral excretion commenced on the 7th day post-challenge, with Ct values of 18.92 and 21.49 for the two puppies displaying clinical symptoms at this stage. In contrast, vaccinated puppies did not exhibit Ct values suggestive of high viral loads. The discrepancy between the lowest Ct value of vaccinated puppies and that of controls was nearly 8 Ct, corresponding to approximately a 2.6 log difference.

#### 3.3.3. Evaluation of the Vaccine’s Immunogenicity

The vaccine generates a robust serological response, indicating significant antibody production. This response is particularly encouraging, as it suggests strong immunity against the targeted pathogen. The levels of antibodies detected in vaccinated individuals surpass expectations, highlighting the vaccine’s effectiveness in eliciting a protective immune response. Such promising results provide confidence in the vaccine’s ability to confer immunity and effectively combat the targeted disease. This is illustrated in the following tables (Table 6 and Table 7).

## 4. Discussion

The objectives of this article are to confirm the circulation of the CPV-2c variant despite the use of vaccines based on the original CPV-2 strain and to highlight the need for a new vaccine targeting the circulating genotypes (2-bc). We aim to develop a new vaccine from a selected Moroccan strain containing the 2-bc variant and to evaluate its safety and efficacy.

Canine parvovirus is extremely contagious, primarily spreading through direct contact with infected feces. Infected dogs can excrete the virus in their feces for several weeks to several months, even after symptoms have resolved [13].

In Morocco, research has confirmed the presence of canine parvovirus (CPV-2) [3,4,5] and identified the co-circulation of CPV-2b and CPV-2c by [14,15]. This finding raises important questions: does the existing vaccine fail to provide adequate protection, and is the development of a new vaccine necessary?

To answer these questions, we employed several approaches, including screening and genotyping canine parvovirus by using real-time PCR, and attempting to isolate the candidate strain to produce a vaccine suitable for the Moroccan context.

The results from the screening and genotyping tests using real-time PCR clearly indicate the presence of multiple CPV variants circulating in Morocco, including CPV-2b and CPV-2c the presence of co-infection has been detected, the co-infections with different CPV-2 variants were previously reported in Italy [14,15]. This indicates a diverse viral population contributing to the challenge of controlling the disease in the region. This has been reported in recent years [14] and demonstrated in this current study

Followed by attempts to isolate the virus on MDCK cells [16]. The “Canivax-parvo” vaccine was produced from an indigenous strain isolated from a symptomatic dog, emphasizing the importance of local strain selection for vaccine development.

Despite vaccination with the current vaccine (CPV-2-ab), the disease remains present, suggesting a lack of protection, mutations in the VP2 gene of CPV-2 variants can alter the virus’s antigenic profile, potentially reducing the efficacy of vaccines designed based on the original virus strain. This highlights the importance of developing new vaccines that target prevalent variants, such as CPV-2c, to ensure effective protection [17].

The relationship between these mutations and vaccine efficacy is crucial. Vaccines function by prompting the immune system to identify and combat the virus based on its specific antigens, mainly the VP2 protein. When the VP2 protein mutates, the antigens presented by the virus can change, making it more difficult for the immune system to recognize the mutated virus. As a result, vaccines designed for the original CPV-2 strain might not offer sufficient protection against new variants with significant VP2 mutations, such as CPV-2b and CPV-2c [17].

In addition, causes of vaccine-related failures include vaccine storage or administration errors, non-compliance with vaccine schedules, and failures in vaccine immunogenicity [18,19]. A 2017 survey of Australian veterinarians’ vaccination protocols found that nearly half of respondents did not comply with the recommended guideline to finish primary vaccination at or after 16 weeks of age [20].

Moreover, the vaccine was produced following the standards and guidelines [21] for good vaccine manufacturing practice using the ”CaPV M/3-2022” vaccine strain which is a CPV-2-bc. Various controls, including inactivation, physico-chemical, and biological assessments, confirmed the high quality of the production.

At the conclusion of the safety study conducted at BIOPHARMA’s animal facility to evaluate “Canivax-parvo”, an adjuvanted inactivated vaccine for preventing canine parvovirus, it was determined that administering two injections three weeks apart (primary vaccination) to eight-week-old puppies via the subcutaneous route did not cause any adverse effects or clinical manifestations attributable to the vaccine. The vaccinated puppies maintained excellent health throughout the observation period. “Canivax-parvo”complies with the safety requirements of the European Pharmacopoeia.

Additionally, the efficacy of the “Canivax-parvo” vaccine, an adjuvanted inactivated vaccine against canine parvovirus made from the Moroccan strain “M3/22”, fully complies with the requirements of the European Pharmacopoeia outlined in chapter 0795 “canine parvovirus vaccine (inactivated)”. The serological results and virulence test demonstrate its satisfactory effectiveness. The vaccine protects vaccinated subjects from the clinical signs of hemorrhagic gastroenteritis, significantly reduces virus excretion compared to unvaccinated controls, and produces a satisfactory serological response with reassuring antibody levels, indicating strong immunity. “Canivax-parvo” provides sufficient efficacy guarantees as required for an inactivated vaccine for the prophylaxis of canine parvovirus.

The vaccine developed from the local strain has demonstrated safety and efficacy in challenge trials. However, the comparison between “Canivax-parvo” and the current vaccines used in Morocco should be accomplished to provide more robust understanding of the cross protection between the autologous vaccines, the commercial vaccines formulated with heterologous strains and the circulating field strains.

## 5. Conclusions

The study provides valuable insights into the prevalence of CPV-2 variants in Morocco, diagnostic challenges, and the development and evaluation of a locally produced vaccine. These findings contribute to the ongoing efforts to combat canine parvovirus and improve the health outcomes of dogs in the region.

## Figures and Tables

**Table 1:** Primers and probes.

Assay	Primer/Probe	Sequence 5′-3′	Specificity
TaqMan assay	CPV-For	AAACAGGAATTAACTATACTAATATATTTA	All types
	CPV-Rev	AAATTTGACCATTTGGATAAACT	
	CPV-Pb	FAM—TGGTCCTTTAACTGCATTAAATAATGTACC—TAMRA	
Type 2a/2b MGB probe assay	CPVa/b-For	AGGAAGATATCCAGAAGGAGATTGGA	All types
	CPVa/b-Rev	CCAATTGGATCTGTTGGTAGCAATACA	
	CPVa-Pb	VIC—CTTCCTGTAACAAATGATA—MGB	Type 2a
	CPVb-Pb	FAM—CTTCCTGTAACAGATGATA—MGB	Type 2b
Type 2b/2c MGB probe assay	CPVb/c-For	GAAGATATCCAGAAGGAGATTGGATTCA	All types
	CPVb/c-Rev	ATGCAGTTAAAGGACCATAAGTATTAAATATATTAGTATAGTTAATTC	
	CPV2b-Pb	FAM—CCTGTAACAGATGATAAT—MGB	Type 2b
	CPV2c-Pb	VIC—CCTGTAACAGAAGATAAT—MGB	Type 2c

CPV: canine parvovirus; For: forward; Rev: reverse; Pb: probe; MGB: minor groove binder; FAM: 6-carboxyfluorescein; VIC: 2′,7′-dimethoxy-4′,5′-dichloro-6-carboxyfluorescein.

**Table 2:** Screening and genotyping real-time PCR results.

	All	Mix a/b		Mix b/c		Conclusion
S.N°	FAM	FAM	VIC	FAM	VIC	
1	27.8	16.94	0	19.89	0	2b
2	32.1	0	0	0	29.96	2c
3	20.8	12.65	0	14.86	0	2b
4	18.09	11.92	0	13.74	0	2b
5	18.76	17.64	0	0	16.75	2bc
6	30.26	18.37	0	19.98	0	2b
7	17.67	16.03	0	0	14.9	2bc
8	11.42	0	0	0	12.99	2c
9	13.47	0	0	0	8.9	2c
10	22	0	0	0	14.94	2c
11	20.2	0	0	0	15.99	2c
Neg extr c	0	0	0	0	0	NEG
Pos extr c	21	0	19.58	26.17	0	2ab
NTC	0	0	0	0	0	NEG

S.N°: sample number; NTC: no template control; NEG: negative; Neg extr c: extraction negative control; Pos extr c: positive extraction control.

**Table 3:** Temperature surveillance of puppies following the challenge.

	Day after Challenge														
	Pup ID	D1	D2	D3	D4	D5	D6	D7	D8	D9	D10	D11	D12	D13	D14
Vaccinated puppies	1	38.4	38.5	38.9	38.6	39	38.9	38.4	38.7	38.1	38.7	38.8	38.4	38.5	38.3
	2	38.6	38.1	38.1	38.5	38.3	38.1	38.6	38.8	38.5	38.5	39	38.3	38.7	38.4
	3	39.1	38.4	39	38	38.4	38.2	38.1	39	38.2	38.4	38	38.5	38.5	38.4
	4	38.2	38.8	38.4	38.3	38.7	39.1	38.7	38.1	38.7	38.4	38.7	38.8	39.3	38.4
	5	38.7	38.4	38.5	38.9	38.2	38.7	38.4	38.7	38	38.3	38.1	38.9	38.2	38.4
Unvaccinated puppies	6	38.1	38.5	39	38.7	38.9	39.7	40.8	41.2	Death	-	-	-	-	-
	7	38.7	38.9	38.4	38.2	39.4	39	39.6	39.9	40.7	41	Death	-	-	-

Pup ID: puppies identification; D: day.

**Table 4:** White blood cell rate per mm^3^ for each puppy.

		Day after Vaccination					Day after Challenge			
	Pup ID	D0	D4	D7	D10	D14	D4	D7	D10	D14
**Vaccinated puppies**	1	14,000	10,000	12,000	13,000	10,500	11,200	11,100	10,500	14,000
	2	15,000	15,000	10,000	15,000	12,300	8000	15,900	16,000	10,000
	3	16,000	15,000	11,000	15,000	13,600	15,000	10,900	14,500	12,000
	4	8000	7000	13,000	8000	11,800	8000	9800	11,000	15,700
	5	10,000	11,000	15,000	16,000	13,000	11,200	10,500	13,800	12,700
**Unvaccinated puppies**	6	12,000	14,000	12,000	10,000	11,500	9800	5600	Death	Death
	7	11,000	13,000	10,000	9000	10,300	14,800	9900	4600	Death

Pup ID: puppies identification; D: day.

**Table 5:** Ct PCR results of rectal swabs taken from puppies after challenge.

	Days Post Challenge					
	Pup ID	D0	D4	D7	D10	D14
**Vaccinated puppies**	1	0	29.2	24.81	23.72	24.13
	2	0	36.17	28.14	30.07	29.08
	3	0	34.01	26.56	26.94	25.65
	4	0	0	24.49	23.61	25.1
	5	0	36.57	27	27.02	24.36
**Unvaccinated Puppies**	6	0	27.74	18.92		
	7	0	0	21.49	19.08	

Pup ID: puppies identification; D: day; Ct: cycle threshold.

**Table 6:** Installation of antibodies inhibiting hemagglutination in vaccinated puppies.

		Days after First Injection					Days after Second Injection		
	Pup ID	D0	D7	D14	D21	D28	D35	D42	D49
**Vaccinated puppies**	1	1/16	1/32	1/128	1/512	1/256	1/128	1/256	1/256
	2	0	1/16	1/128	1/128	1/64	1/256	1/256	1/256
	3	1/4	1/64	1/128	1/128	1/256	1/256	1/128	1/128
	4	1/16	1/64	1/128	1/256	1/256	1/256	1/256	1/256
	5	1/16	1/64	1/256	1/128	1/128	1/256	1/256	1/128
**Unvaccinated puppies**	6	1/8	1/8	1/4	1/16	1/8	1/8	1/8	1/16
	7	0	1/8	1/8	1/8	1/16	1/8	1/8	1/8

Pup ID: puppies identification; D: day.

**Table 7:** Installation of antibodies inhibiting hemagglutination in guinea pigs.

ID Cobaye/IHA Titre							
1	2	3	4	5	6	7	8
1/256	1/512	1/256	1/512	1/256	1/512	1/256	1/512
Average: **1/384**							

ID: identification; IHA: antibodies inhibiting hemagglutination.

## Data Availability

The data presented in this study are available in this article.
